# Why the Stall? Using metabolomics to define the lack of upstream movement of invasive bigheaded carp in the Illinois River

**DOI:** 10.1371/journal.pone.0258150

**Published:** 2021-10-07

**Authors:** Jocelyn A. Curtis-Quick, Alexander V. Ulanov, Zhong Li, John F. Bieber, Emily K. Tucker-Retter, Cory D. Suski

**Affiliations:** 1 Department of Natural Resources and Environmental Sciences, University of Illinois, Urbana, Illinois, United States of America; 2 Metabolomics Lab, Roy J. Carver Biotechnology Center, University of Illinois at Urbana-Champaign, Urbana, Illinois, United States of America; University of Windsor, CANADA

## Abstract

Bigheaded Carp have spread throughout the Mississippi River basin since the 1970s. Little has stopped the spread as carp have the ability to pass through locks and dams, and they are currently approaching the Great Lakes. However, the location of the leading edge in the Illinois River has stalled for over a decade, even though there is no barrier preventing further advancement towards the Great Lakes. Defining why carp are not moving towards the Great Lakes is important for predicting why they might advance in the future. The aim of this study was to test the hypothesis that anthropogenic contaminants in the Illinois River may be playing a role in preventing further upstream movement of Bigheaded Carp. Ninety three livers were collected from carp at several locations between May and October of 2018. Liver samples were analyzed using gas chromatography-mass spectrometry in a targeted metabolite profiling approach. Livers from carp at the leading edge had differences in energy use and metabolism, and suppression of protective mechanisms relative to downstream fish; differences were consistent across time. This body of work provides evidence that water quality is linked to carp movement in the Illinois River. As water quality in this region continues to improve, consideration of this impact on carp spread is essential to protect the Great Lakes.

## Introduction

Understanding the distribution of species has long been a focus of ecological research. The distribution of a species results from a complex, dynamic interaction of biotic and abiotic factors [1, 2] that provide access to resources such as food and habitat, enabling successful survival and reproduction [3], coupled with elements of recruitment, migration, gene flow and chance [4, 5]. Human activities can alter biotic and abiotic factors in the environment and result in changes to the distribution of species. In particular, actions such as changing climatic conditions, habitat destruction, or the introduction of pollutants and/or invasive species can have large impacts on the distributions of native species [6–10]. More importantly, if habitat characteristics become sub-optimal and resources or conditions for survival cannot be met, an organism may expand its range into novel areas in an effort to obtain vital resources and avoid extirpation [11], assuming factors such as physiological tolerance, competition and environmental factors, such as temperature, permit such expansion [12–14]. Thus, a number of biotic and abiotic factors will play a role in defining the range and distribution of a species, and this range can be altered by human-induced changes to the environment.

Invasive species that are introduced outside of their native range represent not only an important ecological problem, but also a unique opportunity to better understand how biotic and abiotic aspects of the environment can influence range and distribution. Introduced species often follow a predictable progression of invasion that includes introduction, establishment and spread [15], and can be capable of rapid range expansion due to the removal of evolutionary limits such as competition, disease/parasites or adaptations to a specific habitat or food source. Invasive species are a problem for receiving ecosystems as they can alter habitats and food webs, increase competition and predation, introduce diseases/pathogens and change ecosystem function [16–19]. Freshwater environments are experiencing a disproportionate decline in biodiversity and ecosystem services relative to other biomes [20–22], and invasive species are believed to be a key factor driving these declines [20, 23, 24]. The removal of an invasive species following the establishment and spread stages is almost impossible, such that it is critical to either prevent the initial transport or introduction of species, or else deter their dispersal beyond the point of initial introduction to limit their impacts [25–27]. To accomplish this goal of deterring the dispersal of invasive species, it is critical that the dynamic interaction of biotic and abiotic factors be understood.

Bigheaded carp (specifically silver carp *Hypophthalmichthys molitrix* and bighead carp *H*. *nobilis*) are a group of non-native fishes currently spreading across the Midwestern United States for which knowledge of distribution and spread could have important management implications. Bigheaded carps were introduced into the US in the 1970s for aquaculture purposes but, shortly after their introduction, individuals escaped into the Mississippi River and began spreading. Bigheaded carp can pass through locks/dams on rivers [28] and are capable of moving great distances. By the 1990s, populations had grown and spread across the Midwest, well beyond their point of introduction. At present, bigheaded carps can be found throughout most of the major river basins in the Midwestern United States (Illinois, Mississippi, Missouri, Ohio, etc.). These carp have had a number of negative consequences for the invaded aquatic systems [29], reducing plankton abundances and causing declines in native teleost numbers and condition [29–33]. Interestingly, despite their propensity to move and spread [34], the invading front of bigheaded carp in the Illinois Waterway has not changed in almost a decade, even though there is no physical barrier preventing further movement. Additionally, the non-physical, electric dispersal barriers located near Romeoville, IL, are approximately 24 km from the leading edge. According to the Asian Carp Monitoring and Rapid Response Plan Summary Reports from 2011 [35] and 2021 [36], the ‘leading edge’ of carp in the Illinois Waterway (i.e., the most upstream location closest to Chicago and the Great Lakes) is the Dresden Island pool, and this has not changed for over 10 years. Thus, while bigheaded carp have demonstrated an alarming proficiency to rapidly spread throughout the Mississippi Basin, some aspect of the Dresden Island pool has halted their range distribution. While populations of native fishes are robust, and even increasing, at locations upstream [37], bigheaded carp have mysteriously not advanced for over 10 years. Defining the mechanisms that have prevented bigheaded carp distribution further upstream is critical for making predictions as to why they might move in the future.

There are a number of possible hypotheses that could explain the lack of upstream expansion of carp in the Illinois Waterway over the past decade (e.g., success of the commercial harvesting program intended to keep population levels low, lack of food or suitable habitat, reduced water quality, presence of noxious stimuli), but one hypothesis in particular has received some degree of empirical support. More specifically, evidence exists to suggest that the upstream movement of carp in the Illinois Waterway has been deterred due to the presence of anthropogenic bioactive compounds (i.e., contaminants) in the water. The movement of fish away from areas of degraded habitat or due to noxious stimuli has been well documented [38–40], and is believed to occur as fish ‘choose’ to avoid inhabiting water with sub-optimal quality and avoid costly upregulation of the stress response [40, 41]. Work by Battaglin et al. (2020) [42] showed that a number of pollutants in the Illinois River, including wastewater indicators, metals, hormones and pharmaceuticals, have a higher concentration upstream of the leading edge of carp relative to downstream, and, based on this abrupt change in water quality upstream of the leading edge of carp, Battaglin et al. (2020) [42] suggested that the presence of contaminants “could represent a barrier to carp range expansion”. Additionally, Jeffrey et al. (2019) [43] used a transcriptomics approach to show that silver carp at the upstream edge in the Illinois Waterway had an upregulation of genes related to contaminant exposure and energy use in livers relative to fish in two downstream populations, again suggesting that contaminants “may represent a non-permanent barrier to silver carp range expansion”. Despite the evidence from these two studies linking contaminants to the lack of upstream movement, the transcriptomics study by Jeffrey et al. (2019) [43] was based on a limited sample size (only 2 collection times, and one collection point only had 2 individuals), and only using one of the two species of bigheaded carp in the Illinois Waterway, bringing the consistency of conclusions across time points and across species into question. In addition the transcriptomics study by Jeffrey et al. (2019) [43] used ribonucleic acid sequencing (RNA-seq) to quantify gene expression differences between upstream and downstream populations, and there are limits to this technique in terms of sensitivity, reproducibility and interpretation [44, 45]. Thus, while evidence exists to suggest that anthropogenic bioactive compounds could be playing a role at preventing the upstream dispersal of carp in the Illinois Waterway, this evidence is limited in space, time and techniques, preventing definitive conclusions.

Based on this background, the overall goal of the current study is to explore the relationship between environmental contaminants and the distribution of carp in the Illinois Waterway. To accomplish this goal, we quantified metabolomics signatures from livers in two species of bigheaded carp collected from a number of time points, both at the leading edge of the population as well as from downstream locations. Metabolomics analyses identifies small molecules (metabolites) within tissue related to various compounds within the water (i.e., assimilation of compounds into the fish), and provides the metabolic products of the assimilated compounds generating a ‘snapshot’ of the processes ongoing within a cell allowing identification of the physiological effects of environmental compounds, [46, 47]. Thus, the use of a metabolomics approach for these analyses allows us to accurately define the phenotypic response of individuals to different environmental conditions, rather than simply quantifying the activity of genes [48, 49]. Furthermore, defining the relationship between water quality and the movement of bigheaded carp is critical given ongoing improvements in the Chicago Area Waterway System (CAWS). The past few decades have seen a pronounced enhancement in the water quality of the CAWS, and a resultant improvement in the fish community [50]. This improvement is expected to continue in the future as Chicago upgrades wastewater treatment facilities [51] and increases capacity to treat effluent with deep reservoir programs and tunnels. Therefore, defining the factors responsible for range expansion in invasive bigheaded carp, or a lack of range expansion in the case of the Illinois Waterway, is key to defining their potential to spread into new areas and negatively impact receiving ecosystems in the face of human-induced environmental changes.

## Materials and methods

### Study sites

Bigheaded carp were collected from the Illinois and Des Plaines Rivers, parts of the Illinois Waterway, from May to November, 2018. The Illinois River is located in Illinois, USA, and is formed through the confluence of the Des Plaines and Kankakee Rivers, and flows to its connection with the Mississippi River. Sampling took place at the leading edge of the bigheaded carp invasion, Rock Run Rookery, a 34 hectare side channel lake in the Dresden Island Pool of the Des Plaines River, located 65 km downstream of Lake Michigan and approximately 24 km from the electric dispersal barriers. Additionally, two downstream sites were sampled; near Starved Rock (in the Starved Rock Pool of the Illinois River) approximately 74 km from the leading edge, and also near Havana on the Illinois River (part of the La Grange reach of the Illinois River) approximately 268 km from the leading edge. The Dresden Island Pool is well documented as being the leading edge site for bigheaded carp, and populations in this pool have not advanced closer to the Great Lakes in almost a decade [36, 52]. Both of the downstream sites have established large carp populations since 2000 [30, 53] such that they are considered to be ‘core’ populations.

### Ethics statement

This study was carried out in strict accordance with protocols approved by the Institutional Animal Care and Use Committee (IACUC) of the University of Illinois (Protocol Numbers: #17118 and #21074). Fish were euthanized via cerebral percussion, and all efforts were made to minimize suffering.

### Sample collection

Fish were collected through a combination of electrofishing and gill and trammel nets set by commercial and agency harvesters [43, 54]. The leading edge site at Rock Run by its very nature, has lower numbers of fish compared to the more established populations downstream [43], and the intent of this study was to collect fish from as far upstream as possible. Collections from areas farther downstream would have likely increased sample sizes, but would not have reflected the true leading edge of the population, and would not have occurred in areas of previously shown to contain elevated water contaminants [42]. Following capture, fish were euthanized via cerebral percussion then total length (TL), mass, and sex were recorded. A portion of liver was removed and placed in microcentrifuge tubes stored either on dry ice or in liquid nitrogen, and then moved to -80° C storage prior to analysis, [43, 54]. In total, 93 liver samples were collected, with 12 harvested from the upstream location.

### Metabolite extraction

In the laboratory, approximately 100 mg of liver tissue was transferred into a microcentrifuge tube (sample weights ranged from 64.7–102.1 mg, with an average weight of 99.2 mg), with care being taken to ensure that all samples remained frozen during weighing to minimize degradation of liver metabolites [55]. Each frozen liver sample received an equal volume of 0.5 mL of homogenizing zirconium oxide beads. Microcentrifuge tubes of liver, solution and beads were then homogenized in a NextAdvance™ Bullet Blender Blue Homogenizer (Model BBX24B) on a frequency setting of 8 for 2 cycles of 5 minutes. Polar and non-polar metabolites were extracted with acetonitrile:isopropanol:water (3:3:2 v/v) and methanol/chloroform (1:2 v/v) solutions respectively. Both extracts were collected and transferred into the same tube, followed by complete dryness under vacuum and resuspended in 1mL of acetonitrile:isopropanol:water (3:3:2 v/v).

### GC/MS

For GC/MS analyses, the aforementioned aqueous extracts were subject to complete dryness by SpeedVac. Dried polar and nonpolar extracts were derivatized with 80 μL methoxyamine hydrochloride (40 mg/mL) for 60 min at 50°C and then with 80 μL MSTFA at 70°C for 120 minutes with a following 2-h incubation at room temperature. Twenty microliters (20 μL) of the internal standard (hentriacontanoic acid, 1 mg/mL) was added to each sample prior to derivatization.

Metabolite profiling analysis was acquired using a GC/MS system (Agilent Inc, CA, USA) consisting of an Agilent 7890 gas chromatograph, an Agilent 5975 MSD and a HP 7683B autosampler. Gas chromatography was performed on a ZB-5MS (60 m × 0.32 mm I.D. and 0.25 μm film thickness) capillary column (Phenomenex Inc, CA, USA). The inlet and MS interface temperatures were 250°C, and the ion source temperature was adjusted to 230°C. An aliquot of 1 μL was injected with the split ratio of 10:1. The helium carrier gas was kept at a constant flow rate of 2 mL/min. The temperature program was: 5 minutes isothermal heating at 70°C, followed by an oven temperature increase of 5°C min-1 to 310°C and a final 10 min at 310°C. The mass spectrometer was operated in positive electron impact mode (EI) at 69.9 eV ionization energy at a scan range of m/z 30–800.

The spectra of all chromatogram peaks were evaluated using the AMDIS 2.71 (NIST, MD, USA) software using a custom-built database (460 unique metabolites). All known artificial peaks were identified and removed prior to data mining. To allow comparison between samples, all data were normalized to the internal standard signal in each chromatogram and the sample weight. The instrument variability was within the standard acceptance limit (5%).

### Statistical analysis

Multivariate statistics were performed using SIMCA-P+ (12.0.0.0, Umetrics, Umea, Sweden) and Metaboanalyst 5.0. Partial least squares discriminant analysis (PLS-DA) and orthogonal partial least squares discriminant analysis (OPLS-DA) were carried out to define significant differences in metabolites temporally, spatially, and between species using pairwise comparisons. Seven-fold cross-validation and 1,000 permutations were used to validate statistical models. Determination of the discriminating metabolites toward the clustering in PLS-DA models was further analyzed using regression coefficient plots with 95% jackknifed confident intervals, where metabolites with Variable Importance for Projection (VIP) values exceeding 1.0 were selected as the metabolite cut-off. Specificity and sensitivity for the putative biomarkers was tested with the use of the Receiver Operating Characteristic (ROC) curve method [56, 57]. False discovery rate (FDR) test was employed to address the “multiple testing problem”. Note that the actual energy content of the liver (e.g., lipids) was not quantified.

## Results

A total of 93 fish were collected during sampling for this study comprised of 40 females, 42 males, and 12 unsexed fish (Table 1). GC/MS metabolite profiling analysis detected 180 compounds, and are discussed below.

**Table 1:** A summary of bigheaded carp (silver carp *Hypophthalmichthys molitrix* and bighead carp *H*. *nobilis)* collection efforts in the Illinois River across three locations (downstream: Havana, Starved Rock and upstream Rock Run Rookery) between May and November of 2018.

	Havana	Starved Rock	Rock Run Rookery
**May**	10	10	
**June**	10	9	7
**July**	9	4	
**August**	10	9	1
**September**		10	
**October**			3
**November**			1
**Total**	39 Silver Carp	42 Silver Carp	12 (9 Bighead & 3 Silver Carp)

**Fig 1:**
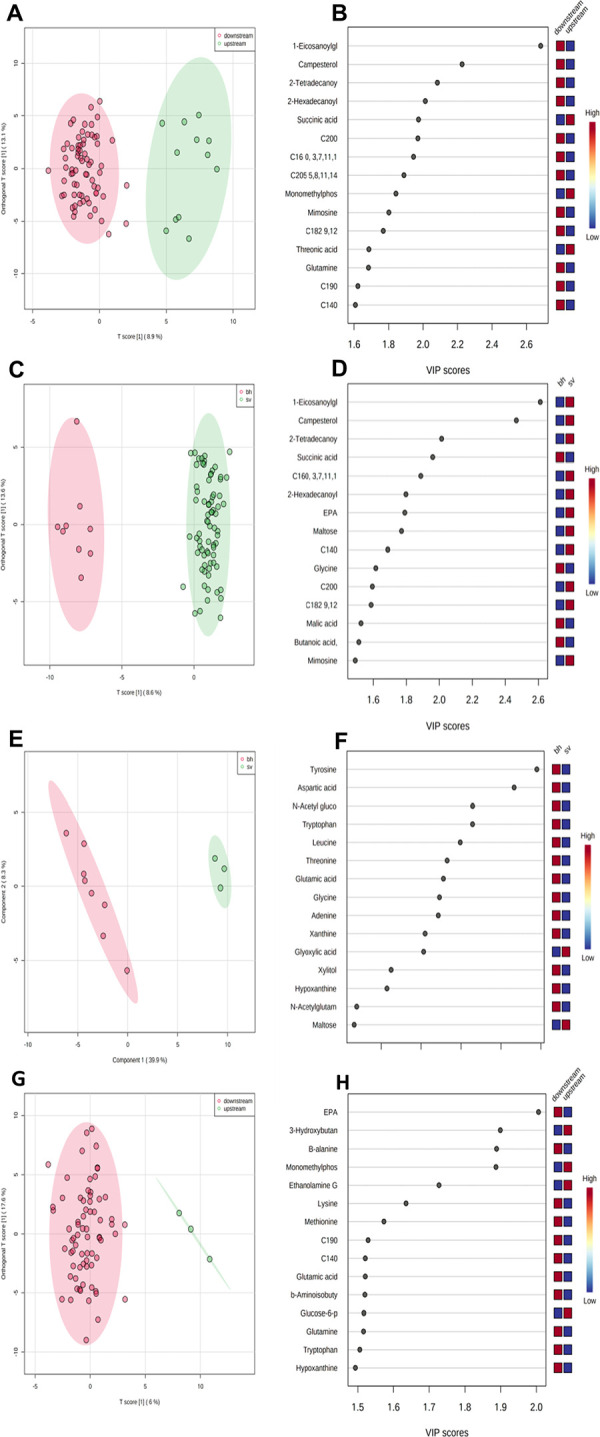
Metabolic patterns in Bigheaded carp with location and species. (A and G) The PLS-DA scores plot’s green and red dots represent upstream and downstream samples, respectively. (A) includes all species (R2 = 0.91; Q2 = 0.69) whereas (G) examines only silvers across location (R2 = 0.514; Q2 = 0.334). In (C) and (E) the green and red dots represent bighead and silver carp, respectively. (C) Examines the metabolites signatures between the Bigheaded carp species (silver carp *Hypophthalmichthys molitrix* and bighead carp *H*. *nobilis*) (R2 = 0.95; Q2 = 0.79). (E) Examines both carp species in the upstream/leading edge, one sample was omitted, fish 93, as it was classed as an outlier (R2 = 0.99; Q2 = 0.84). Figs B, D, F and H in the panel are variable importance in projection (VIP) scores for the top 15 metabolites form the previous PLS-DA plot. Metabolites with values >1.0 were considered important contributors. Higher value of the VIP scores indicates an increased contribution of the metabolite to the location separation. The blue and red boxes indicate whether metabolite concentrations increased (red) or decreased (blue) in livers of fish. Note all analyses excluded fish from May 22^nd^, 2018.

When both species of bigheaded carp were pooled together, liver metabolites signatures from the upstream and downstream location separated from each other (Fig 1A). Of the 180 metabolites searched in the database, the metabolites that had the strongest difference between upstream and downstream locations were Eiosanoyl glycerol and campesterol, with lower concentrations of both compounds exhibited by the upstream samples (Fig 1B and Table 2).

**Table 2:** List of liver metabolites that differed between bigheaded carp (silver carp *Hypophthalmichthys molitrix* and bighead carp *H*. *nobilis)* sampled from leading edge vs. downstream locations within the Illinois River.

	q-value	t-test	Fold Change	AUC
**1-Eicosanoylglycerol**	1.20E-09	1.18E-11	2.6	0.89
**Campesterol**	3.61E-06	7.07E-08	1.8	0.81
**2-Tetradecanoylglycerol**	2.08E-05	6.13E-07	4.0	0.88
**2-Hexadecanoylglycerol**	4.13E-05	1.62E-06	2.0	0.88
**Succinic acid**	4.89E-05	2.74E-06	-1.2	0.84
**C20:0**	4.89E-05	2.88E-06	3.0	0.86
**C16:0, 3,7,11,15-tetramethyl**	5.82E-05	3.99E-06	2.4	0.92
**C20:5 (5,8,11,14,17)**	1.03E-04	8.04E-06	2.7	0.95
**Monomethylphosphate**	1.60E-04	1.41E-05	-0.9	0.82
**Mimosine**	2.33E-04	2.29E-05	1.0	0.87
**C18:2 (9,12)**	3.08E-04	3.32E-05	2.9	0.95
**Threonic acid**	6.64E-04	8.28E-05	-1.9	0.86
**Glutamine**	6.64E-04	8.47E-05	4.1	0.84

When the two species of carp sampled from the Illinois River were compared to each other independent of sample location, results indicated complete separation in liver metabolite signatures mirroring the pattern seen in the all species location analysis (Fig 1C and 1A). Both eiosanoyl glycerol and campesterol were again responsible for contributing to the interspecific differences, with eiosanoyl glycerol and campesterol concentrations reduced in the Bighead carp, which were only found in the upstream/leading edge area (Fig 1D and Table 3).

**Table 3:** Liver metabolites that differed between two species of bigheaded carp (silver carp *Hypophthalmichthys molitrix* and bighead carp *H*. *nobilis)* sampled across locations in the Illinois River.

	q-value	t-test	Fold Change	AUC
**1-Eicosanoylglycerol**	5.63E-14	5.58E-16	-5	0.97
**Campesterol**	5.17E-12	1.02E-13	-4	0.94
**2-Tetradecanoylglycerol**	4.25E-07	1.26E-08	-6	0.94
**Succinic acid**	9.29E-07	3.68E-08	1	0.90
**C16:0, 3,7,11,15-tetramethyl**	2.88E-06	1.42E-07	-2	0.94
**2-Hexadecanoylglycerol**	1.10E-05	6.81E-07	-2	0.91
**EPA**	1.10E-05	7.62E-07	-2	0.93
**Maltose**	1.34E-05	1.06E-06	-3	0.93
**C14:0**	4.38E-05	3.90E-06	-1	0.94

When the two species of bigheaded carp sampled only from the upstream location were compared to each other, analyses revealed that liver metabolites sampled at this location differ significantly between species (Fig 1E). Specifically, tyrosine and aspartic acid were contributing most to the species differences, with both compounds showing reduced concentrations in silver carp (Fig 1F, and Table 4).

**Table 4:** Liver metabolites that differed between two species of bigheaded carp (silver carp *Hypophthalmichthys molitrix* and bighead carp *H*. *nobilis)* sampled only from the leading edge of the population in the Illinois River.

	q-value	t-test	Fold Change	AUC
**Tyrosine**	8.45E-05	8.90E-07	1.7	1
**Aspartic acid**	3.05E-04	6.42E-06	4.4	1
**N-Acetyl glucosamine**	0.001415	5.94E-05	6.2	1
**Tryptophan**	0.001415	5.96E-05	3.3	1
**Leucine**	0.001839	9.68E-05	1.3	1

When the liver metabolites of silver carp were compared across upstream and downstream locations, differences in metabolites were identified. More specifically, eicosapentaenoic acid (EPA) was the strongest contributor to spatial differences within this species, with lower levels present in silver carp sampled from the upstream location relative to conspecifics captured from further downstream. A ROC curve was performed for EPA and showed high discrimination (no overlap in the two distributions) indicating almost a 100% sensitivity and 100% specificity with AUC score of 0.967 (Table 5).

**Table 5:** Liver metabolites that differed in silver carp (*Hypophthalmichthys molitrix)* sampled from leading edge vs. downstream locations within the Illinois Rivers.

	q-value	t-test	Fold Change	AUC
**EPA**	0.0565	7.24E-04	1.7	0.967
**3-Hydroxybutanoic acid**	0.0565	0.00144	-1.4	0.977
**B-Alanine**	0.0565	0.00154	2.1	0.981
**Monomethylphosphate**	0.0565	0.00156	-1.0	0.953
**Ethanolamine GP**	0.0734	0.00398	-1.3	0.934
**Lysine**	0.0734	0.00659	1.4	0.939

**Fig 2:**
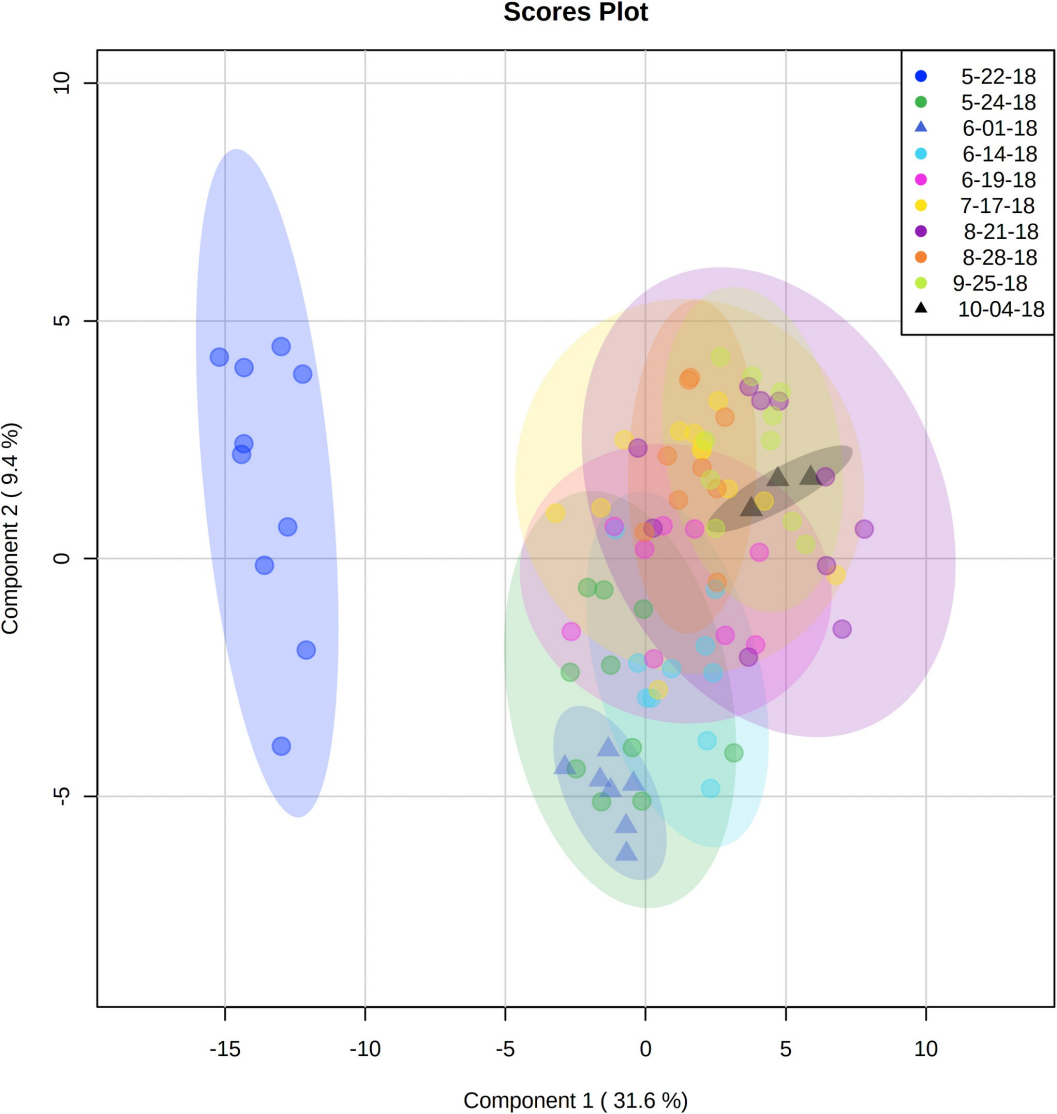
Temporal separation of metabolites in the livers of Bigheaded carp collected from the Illinois river. The legend color denotes the collection date between May and November 2018 and the shape indicates the collection area (upstream/downstream).

To define possible differences across sample dates, a partial least squares discriminant analysis (PLS-DA) of the data matrix was carried out. In general, there was no separation between metabolites across the seven collection points (Fig 2). The lone exception was fish collected from the downstream site of Starved Rock on the 22^nd^ of May 2018, which separated from the other time points relative to other sample dates. Closer inspection of these metabolites indicated that the differences in this sample were due to elevated levels of amino acids (leucine, isoleucine, valine, phenylalanine, lysine, tyrosine and, threonine). The results shown above did not change depending on whether or not this lone sample date was included or excluded from analyses.

## Discussion

Invasive bigheaded carp in the Illinois River showed spatial differences in the metabolite signatures from livers when individuals from the leading edge were compared with fish from downstream, core areas. The two strongest drivers of these differences between locations, obtained from searches of over 180 compounds, related to signatures of Eicosanoyl glycerol acid and campesterol, which were both lower in carp sampled from the upstream area relative to fish from both downstream populations (Fig 1B and Table 2). Eicosanoyl glycerol is a monoacylglyceride, generated from the hydrolysis of a triacylglycerol, and glycerides, and is key to lipid metabolism in fish, and therefore energy use [58–60]. One of the key sites responsible for detoxification in fish is the liver, and past studies looking at lipid concentrations in livers of fish such as Atlantic cod (*Gadus morhua)*, spotted snakehed (*Channa punctatus)* and rainbow trout (*Oncorhynchus mykiss)* all demonstrated a decrease in liver lipids residing polluted environments, which is likely a result of accelerated energy consumption due to increased metabolism from elevated toxins [61–63]. Reduced eicosanoyl glycerol levels seen in bigheaded carp from the leading edge of the Illinois River could therefore be a result of elevated energy use *in situ* due to reductions in water quality [6]. Campesterol is a phytosterol derived from the consumption of plants, and has a role in energy use/expenditure as well as protective mechanisms [64, 65]. For example, a recent study examining streaked prochilod (*Prochilodus lineatus)* showed that fish sampled from locations receiving increased sewage/fecal inputs had lower campesterol concentrations in muscle relative to individuals collected from less polluted environments [66]. Furthermore, the medical literature for humans has also linked higher concentrations of plant sterols such as campesterol to protection against a number of cancers, disease and inflammation, with reduced protection from lower sterol concentration [67, 68]. Note that, when the metabolite signatures of the two species of carp were compared to each other (pooled across locations), again both campesterol and eicosanoly glycerol were reduced in upstream fish (Fig 1D and Table 3). This mirrors the findings of the spatial analysis as silver carp dominated the downstream sites and bighead the upstream leading edge site. Together, reduced concentrations of eicosanoyl glycerol and campesterol in bigheaded carp collected at the leading edge in the Illinois River relative to downstream locations is suggestive of increased energy use in liver, and possibly reduced protection at a cellular level, that could be due to increased xenobiotic exposure from contaminants in the water.

Previous water quality assessments performed in the Illinois River by Battaglin et al. (2020) [42] found concentrations of contaminants and bioactive chemicals (e.g., volatile organic compounds (VOCs), pharmaceuticals, wastewater indicators, hormones and metal) were elevated in samples collected upstream of Rock Run Rookery (i.e., the leading edge of bigheaded carp), with concentrations of many bioactive chemicals declining in downstream locations [42]. In addition, a previous investigation by Jeffrey et al. (2019) [43] found that the gene expression patterns in livers from silver carp collected from Rock Run Rookery showed signatures of increased energy use, and decreased activity of cellular protective mechanisms, relative to silver carp collected from two downstream populations. More importantly, Jeffrey et al. (2019) [43] also found that silver carp collected from Rock Run Rookery at the leading edge displayed upregulation of three members of the ATP-binding cassette proteins, commonly referred to as ABC transporters, which are highly conserved transmembrane transport proteins that confer multixenobiotic-resistance allowing organisms to survive in polluted environments [69–71]. Thus, it is likely that the reduced concentrations of eicosanoyl glycerol acid and campesterol seen in bigheaded carp livers sampled from the leading edge of the population resulted from increased energy use and contaminant exposure due to detoxification, consistent with previous transcriptomics work and environmental monitoring [42, 43]. It should be noted that there are other alternative explanations that should also be investigated. For example, because campesterol is obtained through the diet in fish, it is possible that elevated concentrations of campesterol observed in livers from bigheaded carp in downstream locations are due to differences in phytoplankton communities. Rock Run Rookery, the leading edge of the population, is a backwater area, while downstream sample locations more closely resemble main channel habitats, and phytoplankton communities can differ across these habitat types in rivers [72]. Dense core populations of silver carp may also exert impacts on the abundance of phytoplankton relative to lower density populations at the leading edge, resulting in diet differences [73]. Together, results from the current study demonstrated that bigheaded carp sampled from the ‘leading edge’ of the population front displayed reduced eicosanoyl glycerol and campesterol levels relative to individuals sampled further downstream, which potentially is a result of elevated energy use due to increased levels of contaminants in the Illinois River at the leading edge of the population.

When considered across the May to November sampling points, the metabolomics signatures in the livers of bigheaded carp did not show pronounced temporal variation, indicating a consistency in the metabolic responses to environmental cues. Past work has shown that changes in metabolite signatures can been seen when fish are exposed to contaminants for even short durations [74–76], such that variation in environmental contaminants should have been visible in liver metabolites across the time points sampled here. Both the phytoplankton and zooplankton communities of large rivers in Illinois have been shown to differ both spatially and temporally, impacted by factors such as habitat, flooding and nutrient availability [72, 77]. Despite this variation in food resources, both across seasons and across locations, silver carp and bigheaded carp have been shown to exhibit little temporal variation in nutritional indices when sampled across different regions [78], and previous work showed that silver carp at the leading edge of the Illinois River population were not experiencing impaired nutritional status compared to downstream locations [43], indicating that food resources do not appear to be limiting in these species across either time or space. Water in the upper reaches of the Illinois River contains higher concentrations of containments and flux of bioactive chemicals compared to downstream sites [42], and the plankton and suspended particles that carp feed on can accumulate bioactive chemicals from the water [79–81]. The one exception to this trend is that the 10 fish captured from the Starved Rock in the downstream location on May 22^nd^ showed a puzzling difference in metabolomics signatures. When we examine the cause for the differences in these fish, the notable compounds driving this trend were amino acids (leucine, isoleucine, valine, phenylalanine, lysine, tyrosine and, threonine), likely derived from diet [82]. Because of the extensive harvest efforts ongoing to control populations of carp [83], it is likely that that fish in Starved Rock are recent migrants that are harvested, rather than long-term residents, meaning that the diet-related signature observed on May 22^nd^ were likely accrued from foraging elsewhere. Clearly, 12 of the 13 sampling events resulted in little difference in metabolomics signatures, indicating a stable phenotypic response to environmental properties over time for carp in the Illinois River.

When the livers of silver carp from upstream and downstream locations were examined alone, differences in energy use and contaminant exposure were again noted. More specifically, this species showed a reduction of the metabolite EPA in the livers of upstream fish relative to fish sampled downstream (Fig 1G and Table 5). EPA is an energy source and plays a role carbohydrate metabolism, immunity processes and stress response [59, 84–86]. Previous research on the impacts of pollutants found that fatty acids, including EPA, tend to be reduced when organisms are exposed to toxins [87, 88] and be elevated with diet quality [89, 90]. However, there is a contrasting body of research that has found EPA can increase with water contamination [91]. Thus, the explanation for the decline in EPA in silver carp from the leading edge relative to conspecifics from downstream is not fully understood, but may in part be due to the type of contaminant(s) to which organisms are exposed [91], or food available [92], and should be investigated in future work. Together, results from this study clearly show that silver carp at the leading edge of the Illinois River have reduced levels of EPA relative to the downstream core population, possibly indicative of increased energetic demands as a result of xenobiotic exposure, and further studies are needed explain the mechanism for this finding.

When samples collected from the leading edge location were examined exclusively, differences in lever metabolites were found across the two species of bigheaded carp. More specifically, in the samples from the leading edge, bighead carp showed higher concentrations of the amino acids, tyrosine and aspartic acid relative to silver carp collected from the same location (Fig 1F and Table 4). These amino acids play a role in glucose metabolism as tyrosine is linked to the synthesis of neurotransmitters, and aspartic acid plays a role in fish stamina [93–95]. The metabolic response to contaminants of phylogenetically similar species can vary in fish [96] and other organisms such as crabs and mollusks [97, 98]. While the exact mechanism for this difference in metabolites across species is not known, there are a few possible mechanisms that could explain this trend. For example, the reduction in these metabolites may be linked to an increased energy demand in the livers of silver carp related to the processing of contaminants [94, 95, 99]. Alternatively, gill raker morphology of these two species differs, which enables silver carp to feed on smaller particles relative to bigheaded carp [100, 101] arguably making silver carp a superior competitor [29], such that differences in liver metabolites may be driven by differences in food when these species are found sympatrically. It is important to note, however, that both species of carp were present in low numbers at the leading edge, which is a location known to have reduced water quality relative to downstream locations [102]. Sample sizes for this interspecific comparison are small, and possibly influenced by the limited sampling gear used, such that this trend should be interpreted cautiously. To summarize, we found that, when the upstream samples were compared, the two species of carp displayed differences in metabolites related to amino acids indicating species at the leading edge were exhibiting differences in energetic demands, and perhaps a change in food source, which correlated with xenobiotic exposure.

Results from the current study have a number of potential implications for the management of bigheaded carp in the Illinois River, as well as for population fronts that exist elsewhere (e.g., upstream reaches of the Mississippi and Missouri Rivers). Bigheaded carp are large bodied, strong swimmers [103] that can move up to 60 km/day, and are able to pass through navigation structures and shipping locks. This dispersal ability, combined with their high fecundity and high environmental tolerance, make them difficult, if not impossible, to contain. Spread is a common component of the invasion process, and is believed to occur for a number of possible reasons including the release of evolutionary constraints, climate change and assistance through human transport [28, 104–106]. In opposition to this, fishes have been shown to actively avoid sub-optimal habitats owing to the presence of noxious compounds or adverse stimuli, with one explanation for this habitat avoidance related to a desire to avoid ‘stressful’ conditions that can increase energy consumption that accompanies an upregulation of the stress response [41, 42, 107]. Despite an exceptional dispersal ability, the leading edge of the population front of bigheaded carp in the Illinois River has not advanced further upstream for over a decade. While the mechanism for this lack of spread has not been conclusively defined, one current hypothesis for this phenomenon is that fish at the distribution of bigheaded carp is not expanding upstream due to the active avoidance of anthropogenic bioactive compounds upstream of the leading edge, with carp ‘choosing’ to remain downstream to avoid energetically costly environments associated with contaminant exposure. This hypothesis has been supported with environmental monitoring, as well as by transcriptomics work [43], comparing upstream and downstream locations, as well as metabolomics work from the current study. We encourage investigation of alternative explanations that could contribute to the lack of the upstream expansion of carp within the Illinois River, such as the success of the commercial harvesting program, lack of food (energy reserves) or suitable habitat, noise pollution, or hydrological factors that could deter movements. However, the current data set provides further evidence in support of the hypothesis that water quality may be a factor in preventing movement, demonstrated by indices of increased energy use in liver, one of the primary detoxification site for fish, for fish sampled at the leading edge, which was consistent across seasons and species. This finding is important given the fact that the water quality in Chicago has improved considerably since the implementation of the Clean Water Act and the formation of the Metropolitan Water Reclamation District of Greater Chicago that oversees the management of waste and storm water [108]. These improvements in water quality are evidenced by the increased abundance and diversity of native fish species in the CAWS since 1985 [38, 50]. Furthermore, water quality is expected to improve even more with the completion of the tunnel and reservoir plan (TARP) in 2029 intended to improve the treatment of storm water and minimize sewage discharges, coupled with continuing upgrades to wastewater treatment facilities [51, 109]. This likely improvement in water quality may inadvertently remove compound(s) that are playing a role in deterring the upstream movement of invasive carp, unintentionally facilitating further spread. It is important to emphasize that the conclusions from this study are not intended to promote pollution as a control strategy for carp. Rather, results while correlative, emphasize the need to further explore links between water quality in the Illinois River and the condition and behavior of free-swimming carp to define mechanisms responsible for the decade-long lack of spread.

### Conclusions

An unfortunate hallmark of species invasions is spread, dispersal and movement away from their point of introduction. Bigheaded carp in North America are no exception to this trend, and, since their introduction approximately 40 years ago, they have been spreading and expanding into new areas, undeterred by locks, navigation structures or environmental parameters, making them one of the most prominent invasive species for freshwater environments in North America. The lone exception to this trend has been the invading front within the Illinois River, where the leading edge of the population has stalled within a single pool of the Illinois River for over a decade. Results from this study provide further support for the hypothesis that water quality is impacting carp movement. This is demonstrated by the changes in livers related to increased energy use and decreased protective mechanisms at the cellular level relative to core populations, with these differences remaining consistent across virtually all samples collected over a six month period. Furthermore, these findings were mirrored by the data examining the two sympatric species. This body of work provides evidence in support of additional work to further define relationships between water quality and avoidance behavior for carp in the Illinois River. Such studies will help prevent further upstream spread and minimize the impacts of this group of fishes on aquatic ecosystems in the face of continued environmental change and improvements to water quality in this region.
